# Long read nanopore sequencing for detection of *HLA* and *CYP2D6* variants and haplotypes

**DOI:** 10.12688/f1000research.6037.2

**Published:** 2015-05-20

**Authors:** Ron Ammar, Tara A. Paton, Dax Torti, Adam Shlien, Gary D. Bader

**Affiliations:** 1 The Donnelly Centre, University of Toronto, Toronto, ON, M5S3E1, Canada; 2 The Centre for Applied Genomics, The Hospital for Sick Children, Toronto, ON, M5G0A4, Canada; 3 Department of Laboratory Medicine and Pathobiology, University of Toronto; Program in Genetics and Genome Biology & Department of Paediatric Laboratory Medicine The Hospital for Sick Children, Toronto, ON, M5G1X8, Canada; 4 Department of Computer Science, University of Toronto, Toronto, ON, M5S3G4, Canada; 5 Department of Molecular Genetics, University of Toronto, Toronto, ON, M5S1A8, Canada

**Keywords:** nanopore, DNA, sequencing, haplotype, pharmacogenomics

## Abstract

Haplotypes are often critical for the interpretation of genetic laboratory observations into medically actionable findings. Current massively parallel DNA sequencing technologies produce short sequence reads that are often unable to resolve haplotype information. Phasing short read data typically requires supplemental statistical phasing based on known haplotype structure in the population or parental genotypic data. Here we demonstrate that the MinION nanopore sequencer is capable of producing very long reads to resolve both variants and haplotypes of *HLA-A*, *HLA-B* and *CYP2D6 *genes important in determining patient drug response in sample NA12878 of CEPH/UTAH pedigree 1463, without the need for statistical phasing. Long read data from a single 24-hour nanopore sequencing run was used to reconstruct haplotypes, which were confirmed by HapMap data and statistically phased Complete Genomics and Sequenom genotypes. Our results demonstrate that nanopore sequencing is an emerging standalone technology with potential utility in a clinical environment to aid in medical decision-making.

## Introduction

An important aspect of precision medicine is the study of how genes influence individual response to drug therapies, known as pharmacogenomics (PGx). PGx genotyping impacts the choice of drug dosing in many medical contexts. As an example, in acute lymphoblastic leukemia patients the metabolizer status of thiopurine methyltransferase ( *TPMT*) must be considered when calculating the initial drug dose of mercaptopurine (6-MP) to ensure proper treatment and avoid fatal toxicity ^1, 2^.

PGx data are typically collected by sequencing a small panel of known PGx genes via traditional Sanger sequencing, or targeted genotyping technologies ^3^. Diagnostic labs are also exploring the use of whole genome or exome sequencing (WGS, WES) for PGx. However, existing methods have various limitations, which may lead to adverse drug responses. WGS and WES methods may fail to capture or provide adequate sequence coverage for certain PGx loci. Targeted genotyping approaches, such as Taqman (Life Technologies), Luminex (Luminex Corp.) or Sequenom (Agena Bioscience), can fail to detect novel loss-of-function mutations due to their selective interrogation of predefined genomic loci. In a diagnostic clinic, where results are often required within days of administering diagnostic tests, some existing technologies can delay the return of clinical results. Current massively parallel technologies have high capital costs (ranging from $100,000–$1,000,000) requiring clinical laboratories to purchase and maintain large instruments to perform in-house genotyping. Alternatively, a laboratory can send these PGx samples to a third party service for a fee, but may wait up to several months for a clinical report.

Clinical haplotypes, tightly-linked collections of inherited alleles, that are responsible for a plethora of medical phenotypes including patient drug response are important in many medical sequencing applications. Information about haplotypes, or genotype phase, can be inferred from parental genotypes or genetic pattern frequency in the human population, however, these predictions can be inaccurate if *de novo* or rare haplotypes are encountered in a patient ^4, 5^. Due to the chromosomal distance between alleles, current short read technologies in use for PGx are often unable to resolve haplotype information without supplemental statistical phasing or parental genotypic data.

Recently, Oxford Nanopore Technologies Inc. has developed the MinION, a real time nanopore-based DNA sequencing instrument which is compact, inexpensive and faster than most established DNA sequencing technologies. The time it takes from initiation of library preparation to basecalling the first sequence read is approximately 3 hours and the instrument is capable of detecting long sequence reads in excess of 50kb (according to the manufacturer, ONT). Nanopore-based technology promises major advances in DNA sequencing by offering an inexpensive (e.g. on the order of $1000) pocket-sized device for clinical diagnostics or field experiments.

Here we report nanopore-based sequencing of three clinically relevant PGx genes to identify medically actionable variants and haplotypes without statistical phasing.

## Methods

### PCR amplification

Primer sequences for *HLA-A*, *HLA-B* and *CYP2D6* are available in Table S1. For *CYP2D6*, we designed primers to specifically amplify *CYP2D6* while not amplifying the 94% identical *CYP2D7*. PCR primer specificity was verified using UCSC *in silico* PCR. We used the standard protocol (for fragments up to 8 kb) of the KAPA LongRange HotStart PCR system: 5× KAPALongRange Buffer (without Mg _2_^+^) 1×, MgCl _2_ (25 mM) 1.75 mM, dNTPs (10 mM each dNTP) 0.3 mM, Fwd primer (10 μM) 0.5 μM, Rev primer (10 μM) 0.5 μM 50ng of genomic DNA (1ul of a 50ng/ul preparation), KAPA LongRange HotStart DNA Polymerase (2.5 U/μl) 1.25 U/50 μl, PCR grade water up to 50μl. For *HLA-A* and *HLA-B*, genomic sequence was downloaded from UCSC Browser with common SNPs masked. Primers were designed using Primer3 using parameters of 68°C for optimal annealing temp and 26bp minimum primer length ^6^. PCR cycling conditions were: 94°C for 3 mins, followed by 35 cycles of 94°C for 20 sec, 68°C for 15 sec and 68°C for 5 mins followed by a final step of 72°C for 5 mins and hold at 10°C.

### Oxford Nanopore genomic DNA library preparation

The DNA libraries were prepared using the Oxford Nanopore Genomic DNA Sequencing protocol (SQK-MAP003). 1.5μg of PCR product was used (instead of the suggested 1μg based on improved yield in earlier testing) with equimolar amounts of *CYP2D6*, *HLA-A* and *HLA-B* amplicons in solution. DNA was not fragmented because the PCR amplicons were already at the desired size for sequencing and downstream haplotyping (4–5Kbp; Table S1). In accordance with the protocol, we end-repaired the DNA with the NEBNext end repair module (New England Biolabs, cat. no. E6050) and subsequently dA-tailed the sample using the NEBNext dA-tailing module (New England Biolabs cat. no. E6053), prior to ligation of nanopore-specific adapters. All purifications were accomplished with Agencourt AMPure XP beads (Beckman Coulter Inc., cat. no. A63880). Throughout the library preparation, care was taken not to vortex or vigorously pipette/mix the library to avoid shearing the DNA into smaller fragments.

### Oxford Nanopore MinION sequencing and basecalling

The MinION flowcell (R7.3 flowcell chemistry) was run for 24 hours using the MinKNOW software (v47.3) producing 24,859 fast5 files, corresponding to individual reads from base detection events at specific nanopore channels. Online basecalling was performed using the Metrichor software (v2.23). The MinION outputs 3 reads for each dsDNA molecule that passes through a pore. The leading ssDNA is referred to as “1D template” and its complementary ssDNA strand is the “1D complement”. When both 1D template and 1D complement reads are basecalled, a 2D consensus sequence is determined based on complementarity. All read information was extracted using the python HDF5 package h5py ( http://github.com/h5py/h5py). We observed 19,655 1D template reads, 9,584 1D complement reads and 7,540 2D reads. The mean lengths were 2,693bp for 1D template, 2,706bp for 1D complement and 3,486bp for 2D consensus.

### Read alignment

Existing massively parallel sequencing instruments, such as the Illumina HiSeq 2500, produce accurate short reads typically up to ~250bp in length. These sequencers can produce hundreds of millions of reads which need to be rapidly aligned to a reference genome. Current computational methods for accurate alignment of these reads, including BWA ^7^ and Bowtie2 ^8^ are based on the Burrows-Wheeler Transform FM index, and they are designed to align short reads with minimal variation to a reference assembly. BWT-FM methods are insufficiently sensitive to align much longer reads with higher error rates ^9^. These long reads, generated by single molecule sequencers such as the Oxford Nanopore MinION or the Pacific Biosciences RS II, have a significantly higher error rate, enriched for insertions or deletions (indels) rather than substitutions ^9^. Mapping of these reads is best suited to aligners that were originally designed for whole genome alignments, such as LAST ^10^. We chose to use BLASR, originally developed for the Pacific Biosciences system, to align our data, because it was designed to align long error-prone reads rather than genomes ^9^.

All reads were aligned to the human genome reference assembly GRCh37.p13 (hg19) using default BLASR parameters (gap open penalty = ten, gap extension penalty = zero, minimum seed length = 12). The use of other parameters, such as a gap-open penalty of zero (with default gap extension penalty = zero) did not alter the results, even though it may be expected to do so given the prevalence of indels expected in single molecule nanopore sequencing. The majority of successfully aligned long read fragments were obtained from 2D basecalls ( Table 1), and these were of higher quality because they are consensus reads constructed from corresponding 1D template and complement. For the final alignment data, for each separate read event (1D template, 1D complement and 2D consensus), we selected the 2D read if it was available. Since the 1D reads typically had lower mapping accuracy and significantly shorter aligned fragments ( Table 1), these were not included in our variant or haplotype calling analysis.

To repeat our findings, we performed the sequencing experiment using the newest Oxford Nanopore Genomic DNA Sequencing protocol (SQK-MAP004). The library was sequenced on 3 fresh R7.3 flow cells, only one of which produced a significant number of reads. The 2D consensus reads generated using the newer protocol were longer on average (3885.3bp compared to 2952.3bp, see Table 1) suggesting advances in basecalling accuracy since our initial experiments. However, yield was significantly lower with a total of 885 aligned reads for all targeted loci, which did not enable us to make accurate genotyping calls.

**Table 1.** Basecall and read mapping statistics.

Read Type	Number of reads	Mean Length, unaligned (bp)	Number of reads aligned	% of reads aligned	Mean Length, aligned fragment (bp)	Mean substitution frequency	Mean deletion frequency	Mean insertion frequency	Mean mapping accuracy (for each read [match bp/total bp])
1D template	19655	2693.7	3793	19.3%	872.8	8.9%	13.9%	5.7%	71.5%
1D complement	9584	2705.7	2717	28.3%	292.7	7.3%	15.4%	4.1%	73.2%
2D consensus	7540	3486.3	4761	63.1%	2952.3	7.0%	13.3%	5.3%	74.3%

**Figure 1.**
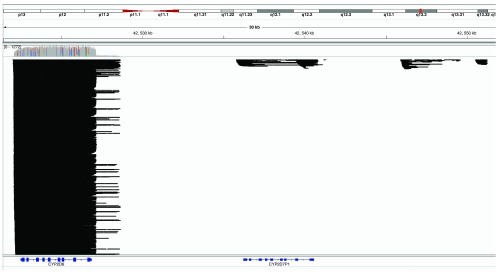
Integrate Genomics Viewer (IGV) diagram of MinION reads aligned to the *CYP2D* locus on chromosome 22 from 42,521,411 to 42,552,401. The majority of reads aligned across the entire length of *CYP2D6* as was expected by selective PCR amplification. Downstream, an insignificant number of read fragments aligned to *CYP2D7* and *CYP2D8* ( *2D8* is located from 42,545,874 to 42,551,097; exon-intron diagram not shown in gene annotation track). Due to the extremely high coverage at *CYP2D6*, not all reads are shown in this pileup diagram.

Finally, we performed two separate alignments depending on our desired sequencing application: a) gene targets with highly similar nearby genes; and b) highly polymorphic gene targets. The first analysis only selected the single best alignment for each long read. This was critical for the gene *CYP2D6* because the *CYP2D* locus on chromosome 22 harbors two paralogous pseudogenes *CYP2D7* and *CYP2D8P* ^11^. In particular, *CYP2D6* and *CYP2D7* are highly similar (94% identity, BLAST E-value = 0.0) and are positioned in tandem on the chromosome. By allowing reads to only map to a single best hit, we were able to verify that the PCR selectively amplified *CYP2D6* and not nearby related genes (see coverage in Figure 1). Our second analysis was performed due to the high degree of polymorphism in the MHC locus on chromosome 6. As part of the MHC haplotype project ^12^, multiple reference contigs for this highly variable region are included in the GRCh37 reference assembly as indicated in the release notes ( http://www.ncbi.nlm.nih.gov/genome/guide/human/release_notes.html). Since long reads from the NA12878 *HLA-A* and *HLA-B* genes mapped to different chromosome 6 reference haplotype contigs, by allowing multiple alignments to the reference (up to 10) for each read, we could gather all reads for a single gene in a single pileup to any of the eight *HLA-A/B* loci to generate a consensus sequence. For this study, we used the reference chromosome 6 contig NC_000006.11 (not the MHC haplotype project contigs).

### Variant detection and haplotype identification

Due to the long reads, high error rates and continuously evolving error profile of the MinION basecalls at this early stage of technology roll out, variant callers such as the Genome Analysis Toolkit’s UnifiedGenotyper or HaplotypeCaller ^13^ were unable to identify variants or haplotypes in the MinION sequence data during our trials. Variant and haplotype level information, however, was readily accessible based on coverage of aligned reads, which we extracted using SAMTools via the Pysam wrapper ( http://github.com/pysam-developers/pysam) ^14^.

Mean coverage was 1236.4× for *CYP2D6* (single best hit alignment), 785.5× for *HLA-A* (multi-hit alignment) and 1416.3× for *HLA-B* (multi-hit alignment).

Variants were detected using a naïve threshold requiring 1/3 of reads to contain the variant genotype at that position. While this was effective for substitution detection, we are likely to detect many false positive deletions due to the high deletion error rate ( Table 1). Haplotype proportions were identified by interrogating clinical marker positions ( Table S2) across all reads aligned to a particular gene to establish the proportion of reads corresponding to each haplotype. Pharmacogenomic haplotypes were verified by comparison to diagnostic data sets (see below) using the MedSavant software ( www.medsavant.com; manuscript in preparation) with the pharmacogenomics app that we developed. The PGx app ** interprets human pharmacogenomic variants with medically actionable output based on published guidelines established by the Clinical Pharmacogenetics Implementation Consortium (CPIC) and the Pharmacogenomics Knowledgebase ( www.pharmgkb.org).

### Validation of genotypes by Complete Genomics, and clinical diagnostic Sequenom MassARRAY and qPCR

Complete Genomics WGS data for NA12878 were obtained from the public 69 genomes project (CG analysis pipeline version 2.0.0; http://www.completegenomics.com/public-data/69-Genomes/) ^15^.

10ng of genomic DNA from NA12878 was genotyped for 36 SNP, indel and copy number variants for *CYP2D6* using the iPLEX ^®^ ADME CYP2D6 Panel v1.0, developed by Assays by Agena (formerly Sequenom) on the MassARRAY4 System. Haplotype assignment and copy number determination was done using Typer software version 4.0 (Agena Biosciences).

**Figure S1 (Updated).**
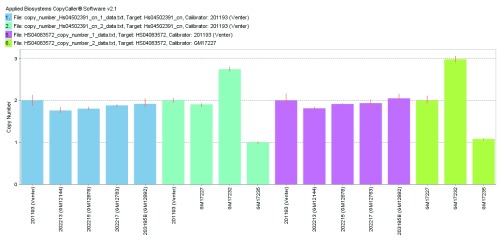
CopyCaller software analysis of data from Taqman copy number assays Hs04502391_cn and Hs04083572_cn. *CYP2D6* from sample NA12878 was observed to be diploid. Samples known to be 1 copy or greater than 2 copies for CYP2D6 (GM17235 and GM17232 respectively ^28^) were run for the two Taqman copy number assays as well.

In parallel, copy number estimation of *CYP2D6* was performed using the Taqman copy number assays Hs04502391_cn and Hs04083572_cn (Life Technologies) using the manufacturer’s recommended protocol ( Figure S1). The assay was performed in quadruplicate on 10ng genomic DNA for each sample in a 96-well plate. The 10μL reaction mix consisted of 5μL 2× Taqman Genotyping Master Mix (Life Technologies), 0.5μL of 20X copy number assay (described above), 0.5μL TaqMan RNAse P Copy Number Reference Assay (Life Technologies cat. no. 4403326), 2μL water and 2μL of 5ng/μL genomic DNA. Cycling conditions for the reaction were 95°C for 10 min, followed by 40 cycles of 95°C for 15 sec and 60°C for 1 min. Samples were analyzed using the ViiA™ 7 Real-Time PCR System (Life Technologies) and analyzed using CopyCaller Software (Life Technologies). The HuRef sample ( http://huref.jcvi.org) was used as a 2-copy calibrator sample.

### Validation of haplotypes by statistical phasing and HapMap

Complete Genomics WGS and Sequenom MassARRAY genotypes were statistically phased using the BEAGLE software (v4.0) and the 1000 Genomes Project phase 3 reference panel ( http://faculty.washington.edu/browning/beagle/beagle.html) ^4^. For the *HLA-A/B* genes, phase information was obtained from the HapMap phase 2 data ( http://hapmap.ncbi.nlm.nih.gov/downloads/index.html.en) ^16^. *HLA-A/B* alleles were determined using the GATK HLACaller software package ( http://gatkforums.broadinstitute.org/discussion/65/hla-caller).

## Results

To evaluate the MinION for diagnostic PGx sequencing, we selectively amplified and sequenced the genes *CYP2D6*, *HLA-A*, and *HLA-B* from the CEPH/UTAH pedigree 1463 sample NA12878. *CYP2D6* is a pharmacogenetically vital cytochrome P450 gene because it encodes a protein responsible for metabolism of 20% of clinically used drugs ^11^. The diagnostic relevance of *2D6* is derived from its significant polymorphism which contributes to dramatic inter-individual variability in enzyme activity ^11^. Also important are the *HLA* genes which are clinically relevant for solid organ transplantation and accurate dosing of abacavir, allopurinol and carbamazepine, used to treat HIV/AIDS, hyperuricemia and seizure disorders, respectively ^17– 19^. The *HLA* genes are among the most polymorphic loci in the human genome, making their sequencing and confident typing difficult with current short read DNA sequencing methods. These three genes were also chosen for sequencing due to their length (4–5Kbp), which did not require long range PCR amplification methods.

**Figure 2.**
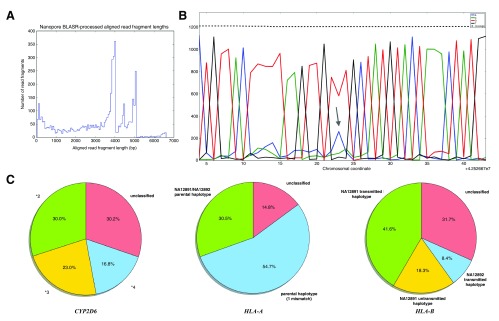
**A**. Length distribution of aligned reads. 4–5Kb reads represent full-length PCR amplicons. Slightly smaller fragments were likely byproducts of shearing during DNA handling in the experimental protocol. **B**. With depth of coverage of ~1000× for each of the genes, many chromosomal positions were called with 70–90% consensus. This is a short window of aligned reads for the *4 locus of CYP2D6 with over 1200× coverage. The heterozygous *4 allele rs1065852 is indicated with the arrow. **C**. Proportions of haplotypes of *CYP2D6, HLA-A* and *HLA-B* when directly measured from individual reads spanning all haplotype markers.

PCR amplicons of these three genes from NA12878 (CEPH/Utah Pedigree 1463) were sequenced on the MinION instrument yielding 19655 read events. Each read event could be basecalled in multiple forms, as a template or complement strand (1D) or as a consensus of the two (2D), and we obtained 36779 1D and 2D reads in total. For the purpose of diagnostic evaluation, we chose to align only the consensus 2D reads due to their lower error rate and extended length ( Table 1; see Methods). Reads were aligned to the human genome (GRCh37), with an abundance of aligned reads 4–5Kb in length representing full-length PCR amplicons. As well, smaller aligned read fragments were observed, some of which are speculated to be by-products of shearing during experimental DNA handling ( Figure 2A, Table S1).

With depth of coverage of ~1000× for each of the genes, many chromosomal positions were called with 70–90% consensus, demonstrating that as coverage of loci increases on the MinION, confidence improves with regard to specific base calls ( Figure 2B). While the MinION basecalls are emitted with a comparatively high error rate ( Table 1), the majority of errors appear to be randomly distributed across the length of the reads, which is why increasing coverage can yield a consensus that matches variant calls from existing sequencing and genotyping platforms such as Illumina, Complete Genomics and Sequenom.

MinION-called variants and haplotypes were validated against statistically phased genotypes from multiple platforms including Complete Genomics and Sequenom MassARRAY (see Methods). Based on the statistically phased genotypes, we determined that NA12878 possesses both the *3 and *4 loss-of-function alleles for *CYP2D6*, and this *3/*4 diplotype is interpreted as reduced metabolism of drugs such as codeine (an opiate) and olanzapine (an atypical antipsychotic) ^20– 22^.

*CYP2D6* haplotype proportions in MinION data were identified by interrogating clinical marker positions across all aligned reads to establish the proportion of reads corresponding to each PGx haplotype ( Table S2). Only reads spanning all clinical markers were included (n = 404), so that haplotypes could be measured by linkage of markers on a single DNA molecule. The MinION data confirmed the statistically-phased haplotypes by direct interrogation of markers from individual reads ( Figure 2C). However, we also observed a prominent *2 haplotype, which we could not account for given that our Sequenom MassARRAY and qPCR results indicated that the *CYP2D6* locus was diploid (no copy number variation) and could only correspond to a *3/*4 diplotype. To determine whether the *2 haplotype could arise from mismatched *CYP2D7* DNA, which was not supposed to be PCR amplified (see Methods), we interrogated four positions with different bases between *CYP2D6* and *CYP2D7* reference sequences and found that all reads corresponded to *CYP2D6* ( Supplementary File S1). Finally, we hypothesized that the *2 haplotype might arise due to duplexes forming between *3 and *4 oligonucleotides during PCR (effectively outcompeting the primer binding during the annealing step), but this was ruled out by identifying the *2 haplotype using only 1D reads. It is possible that this *2 haplotype arose either due to early cycle template switching during PCR or sample contamination ^23^. Also, the relative proportion of haplotypes was potentially skewed by the PCR amplification step.

The *HLA-A* and *HLA-B* haplotypes were determined in the same way as the *CYP2D6* haplotypes, using predefined markers from the HapMap project. In *HLA-A* (only spanning reads, n = 203), the most abundant haplotype matched the transmitted haplotypes of the parents NA12891 and NA12892, which both transmitted an identical haplotype ( Figure 2C). Accounting for the errors in MinION sequencing, when allowing for a single mismatch in the haplotype, ~85% of reads confirm the NA12878 diplotype. In *HLA-B* (only spanning reads, n = 202), the majority of reads corresponded to the transmitted and untransmitted haplotypes of the parent NA12891, with only 8.4% of reads corresponding to the transmitted haplotype of parent NA12892 ( Figure 2C). This could be a result of potential contamination suspected earlier in described above with *CYP2D6*. As suggested for CYP2D6, the relative proportion of HLA haplotypes was likely also affected by PCR bias during amplification. *HLA* alleles were called with 4-digit resolution using the GATK HLACaller, but due to the high error rates of nanopore reads, HLA alleles did not match with alleles called using HapMap data ( Table S3) ^24^.

Nanopore reads and alignments File R7-3_pgx_metrichor2-23_1D.fastq: 1D template reads from MinION device
File R7-3_pgx_metrichor2-23_1D_complement.fastq: 1D complement reads from MinION device
File R7-3_pgx_metrichor2-23_2D.fastq: 2D consensus reads from MinION device
File R7-3_pgx_metrichor2-23_only_2D_best_hg19.sort.bam (and .bai index): Single best BLASR alignments of 2D reads
File R7-3_pgx_metrichor2-23_only_2D_hg19.sort.bam (and .bai index): Up to top 10 BLASR alignments of 2D reads Click here for additional data file. Copyright: © 2015 Ammar R et al. 2015 Data associated with the article are available under the terms of the Creative Commons Zero "No rights reserved" data waiver (CC0 1.0 Public domain dedication).

## Conclusions and discussion

Phasing of genotypes is critical to prevent misinterpretation of PGx variants. The importance of correct phasing of PGx genotypes is illustrated with the gene *TPMT*, which plays a critical role in the metabolism of thiopurine, a drug used to treat acute lymphoblastic leukemia. In a recent study ^25^, an individual was reported to have a TPMT *3B/*3C diplotype, based on observed heterozygous genotypes for the rs1142345 and rs1800460 variants, but this was a misinterpretation due to faulty haplotyping and *1/*3A is the correct diplotype. The rs1800460 variant is present in both *3A and *3B haplotypes while the rs1142345 variant is present in both *3A and *3C haplotypes. As a result, it was possible for the individual to have a *1/*3A diplotype or a *3B/*3C diplotype. Clinically, a *1/*3A diplotype corresponds to an intermediate metabolizer, requiring a 30–70% reduction in thiopurine dose, while a *3B/*3C diplotype corresponds to a poor metabolizer with a 90% reduction in dose ^2^. An individual who receives a standard dose and is a poor metabolizer can experience fatal toxicity, while a low dose for a normal metabolizer can lead to disease progression. Clinical trials have demonstrated the medical importance of TPMT haplotyping in treatment of myeloid leukemias and non-malignant immunologic disorders ^2, 26^.

Long sequence reads aid haplotype identification by determining which genetic variants are in phase (i.e. on the same DNA strand). If the *TPMT* genotypes from the example above were sequenced using nanopore-based long read technology, the *1/*3A diplotype would likely be called correctly (note that rs1142345 and rs1800460 are only 8,310bp apart).

While nanopore sequencing with the MinION is demonstrably error-prone in its current stage of development, we assert that this technology holds promise for clinical applications because accurate consensus sequences can be built with sufficient coverage given the high number of reads generated. As well, we have been able to successfully call haplotypes from long reads *de novo* in the absence of parental haplotypes or statistical phasing. The MinION device produced sufficiently long mappable reads to phase all variants in the loci examined. As error rates on the MinION decrease, we can expect to deconvolute these data into more accurate diplotypes with less noise and will be able to measure how much multi-sample multiplexing can be supported by a single run.

According to the CPIC, 63 genes and 132 drugs have guidelines for pharmacogenomic status ( http://www.pharmgkb.org/cpic/pairs), and this list is constantly expanding. With increasing guidelines and demands for PGx in the clinic, affordable and rapid nanopore sequencing may hold great utility.

## Data availability

The data referenced by this article are under copyright with the following copyright statement: Copyright: © 2015 Ammar R et al.

Data associated with the article are available under the terms of the Creative Commons Zero "No rights reserved" data waiver (CC0 1.0 Public domain dedication). 

figshare: Nanopore reads and alignments, doi: http://dx.doi.org/10.6084/m9.figshare.1289717 ^27^

Raw nanopore reads and alignment files are available at the NCBI Sequence Read Archive, accession SRP051851 ( http://www.ncbi.nlm.nih.gov/sra/?term=SRP051851).

## Figures and Tables

**Table S1.** Primer sequences and amplicon lengths for *HLA-A*, *HLA-B* and *CYP2D6*.

Oxford Nanopore PGx primer list				
Gene	Primer Name	Primer seq	Chromosomal Coordinates	Amplicon Size
CYP2D6	CYP2D6-2F	TAGCTCCCTGACGCCATGATTTGTCTT	chr22:42,522,077-42,527,144	5,067 bp
CYP2D6	CYP2D6-2R	CCTGGTTATCCCAGAAGGCTTTGCAG		
HLA-A	HLAA-2F	AGAAGAGTCCAGGTGGACAGGTAAGGAGTG	chr6:29,909,854-29,913,805	3,951 bp
HLA-A	HLAA-2R	TTCTACTGAAGGGCCAAGGACAATGGAG		
HLA-B	HLAB-2F	TGGATTCAGCACCAAGATCACTAGAACCAG	chr6:31,321,279-31,325,303	4,024 bp
HLA-B	HLAB-2R	GTCTCTCCCTGGTTTCCACAGACAGATCCT		

**Table S2.** Haplotype translation table for *CYP2D6*.

Haplotype Id	CYP2D6	rs1065852	rs28371706	rs5030655	rs3892097	rs35742686	rs5030656	rs16947	rs28371725	rs1135840
PA165816576	*1	G	G	A	C	T	CTT	G	C	C
PA165816577	*2	G	G	A	C	T	CTT	A	C	G
PA165816578	*3	G	G	A	C	-	CTT	G	C	C
PA165816579	*4	A	G	A	T	T	CTT	G	C	G
PA165948092	*5	-	-	-	-	-	-	-	-	-
PA165816581	*6	G	G	-	C	T	CTT	G	C	C
PA165948317	*9	G	G	A	C	T	-	G	C	C
PA165816582	*10	A	G	A	C	T	CTT	G	C	G
PA165816583	*17	G	A	A	C	T	CTT	A	C	G
PA165816584	*41	G	G	A	C	T	CTT	A	T	G

**Table S3.** *HLA* alleles called with 4-digit resolution using the GATK HLACaller.

Locus	HapMap A1	HapMap A2	Nanopore A1	Nanopore A2
HLA-A	0101	1101	0132	0312
HLA-B	0801	5601	0765	5510

## Supplementary File S1

BLAST alignment of *CYP2D6* (bottom sequence) and *CYP2D7* (top sequence). These genes have 94% identity (E-value = 0.0). To determine if our reads were generated from *CYP2D7* reads, we interrogated reads at the paralogous positions circled in red.

## Supplementary File S2

Detailed responses to reviewers' comments.
