# Supplementary material for: Long read nanopore sequencing for detection of HLA and CYP2D6 variants and haplotypes
Source: F1000Res. 2015 May 20;4:17. Originally published 2015 Jan 21. [Version 2] doi: 10.12688/f1000research.6037.2 (PMC4392832; doi:10.12688/f1000research.6037.2)
Supplement: Supplementary file 2 [file f1000research-4-7015-s0001.tgz › e9dbfd22-8053-4fd6-ad52-3b107ebd9b22.docx]

Dear reviewers,

We would like to thank you for your thorough and useful review of our manuscript “*Long read nanopore sequencing for detection of HLA and CYP2D6 variants and haplotypes*”. Please find attached our point-by-point response.

Both of you suggested we reproduce our results using the newest Oxford Nanopore Technology kits and pipeline. Therefore, we performed the sequencing experiment using the newest Oxford Nanopore Genomic DNA Sequencing protocol (SQK-MAP004). The library was sequenced on 3 fresh R7.3 flow cells, only one of which produced a significant number of reads (just over 1000 reads from the other two flow cells). As anticipated, the 2D consensus reads generated using the newer protocol were longer on average suggesting advances in basecalling accuracy since our initial experiments. Unfortunately, yield was significantly lower with a total of 885 aligned reads for all targeted loci (including our 3 genes-of-interest and the nanopore controls), which did not enable us to make accurate genotyping calls. These new read data have been deposited in the NCBI Sequence Read Archive along with our other data.

Specific points are addressed below.

Thank you again for your feedback on our manuscript.

Sincerely,

Ron Ammar and Gary Bader on behalf of the authors.

**Reviewers’ Comments**

**Reviewer #1 (Martin Kennedy):**

**Q1**

Methods

Long PCR has been widely used for specific amplification of CYP2D6, with reaction conditions and primer sequences well established. It is not clear why the authors designed their own primers for this task, or how these novel primers were validated. This should be spelt out more clearly.

**A1**

We designed primers to ensure specificity to *CYP2D6* with no amplification of *CYP2D7*. Full PCR cycling details have been added to the methods section.

**Q2**

It would have been useful to have non-diploid control samples for the CYP2D6 CNV assay – for example a haploid (CYP2D6*5) case or multicopy case, to provide confidence that the assay was working as expected. This is relevant because of the question raised by the *2 haplotype which shows up in the MinIon analysis of NA12878.

**A2**

We have amended Figure S1 to show the controls for CYP2D6*5 (GM17235) and CYP2D6 2N (GM17232) whose copy number for CYP2D6 was validated by Pratt et al 2010. For GM17235 and GM17232, the HuRef sample was the 2-copy calibrator for the Hs04083572_cn assay while the 2-copy calibrator for Hs04502391_cn was GM17227 (Pratt et al 2010).

**Q3**

Results

Page 6, first para: This description of the possible origins of the mystery CYP2D6*2 haplotype needs some editing for improved clarity. Not clear what is meant by “*3 and *4 duplexes forming”. Also not clear what would cause PCR biases alluded to in the last sentence of this paragraph (and of the following paragraph).

**A3**

We have edited this statement for improved clarity.

**Q4**

Typos/suggested edits:

Abstract

MinIOn > MinIon

Should refer to NA12878 as “reference sample” rather than just “sample”

Suggest sentence be modified thus for clarity: “…statistically phased genotype data from Complete Genomics and Sequenom.”

Suggest delete “Standalone” in penultimate line.

Introduction

Suggest this sentence be changed: However, existing methods have various limitations, which may lead to adverse drug responses. > However, existing methods have various limitations, which may lead to failure to detect variants of pharmacogenetic significance.

Methods

Page 3, 2nd para: indels expects > indels expected

Page 4 para 2 – clarify “The HuRef sample…”

Figure 1 title: Integrate > Integrative

Results

Page 5, para 4. First sentence should read thus, for improved clarity: “CYP2D6 haplotype proportions in MinIon data were identified by interrogating clinical marker positions…”

Supp File S1

It would be helpful to indicate which of the sequences is CYP2D6 and which is CYP2D7.

**A4**

We have made all suggested corrections to the manuscript where applicable.

**Reviewer #2 (Thomas Hoenen):**

**Q1**

In order to address the concern that the manuscript is based on a single MinION run, and given the nature of the MAP program, it should be no problem for the authors to repeat the experiment with another sample, and while doing so specifically address the concerns of Dr. Kennedy. In addition, the authors should strive to improve accessibility to a wider audience wherever possible. Finally, it would be helpful to include additional experimental details that are currently missing.

**A1**

We appreciate this suggestion. The experiment was repeated using the newest Oxford Nanopore Technology kits and pipelines as well as multiple fresh flow cells. Unfortunately, as explained above, there was very low combined yield for the three flow cells that were run. We attribute this to variability that we’ve experienced with flow cells as the MinION technology continues to develop. Evaluation of these data has been appended to our manuscript, and the sequence reads were deposited in the NCBI Sequence Read Archive.

**Q2**

PCR cycling conditions should be provided.

**A2**

Full PCR cycling details have been added to the methods section.

**Q3**

The authors refer to the SQK-MAP003 sequencing protocol. As far as I am aware, Oxford Nanotechnologies does not make the detailed protocol available to people outside the MAP program, although there have been indications that a non-technical version of the protocol will be made available for publication purposes. The authors should reference such a protocol (including a link) as soon as possible, and approach ONT about making it available to the general public, if this hasn’t happened already.

**A3**

Unfortunately, as part of the Minion Access Program agreement, at this time we are unable to reveal the details of Oxford Nanopore Technology’s proprietary protocols. These will most likely become publicly available via the company’s website once the technology is commercially available to research labs.

**Q4**

Did the authors perform a PCR purification prior to the library preparation? If so, what was the volume/ratio of Agencourt beads to sample?

**A4**

In accordance with the library preparation protocol, the purification step was part of the first step of the library preparation. The ratio of Agencourt beads to sample was 1x by volume.

**Q5**

How did the authors extract reads from the fast5 files? Did they use poretools, or another tool (which should be referenced)? In general, providing more details about the exact bioinformatics workflow would be helpful.

**A5**

All reads were extracted using h5py, a python library for processing HDF files. While poretools is now available, at the time of our initial analysis it was not publicly available. It would likely replace our scripts if we were to start the study today.

**Q6**

What was the rationale for the cut-off of 1/3 of reads for variant calling?

**A6**

This threshold was chosen because typically heterozygous calls are detectable at roughly 1/2 of reads at a specific position. Since the nanopore sequencer error rate is higher than standard NGS platforms, we reduced that threshold to 1/3.

**Q7**

It seems odd that the length of the aligned fragments from the 1D reads is so much shorter than the read length. In our hands (using a similar approach on ~2 kB PCR products amplified from virus genomes, albeit with a later chemistry/protocol version (SQK-MAP004) and using LAST for the alignment) we get much longer average alignments (92% for 2D reads, 82% for template reads, and 85% for complement reads, vs. 85%, 23% and 11% reported by the authors). This could either indicate significant advances in base-calling accuracy since the authors performed their experiments, or that their alignment is suboptimal. It might be very interesting to see whether the authors can get longer alignments using LAST or other alignment softwares in their workflow.

It would be very helpful if the authors could repeat the experiment using the newest chemistry/protocol/software versions, which have changed considerably over the last months. At the same time this would allow them to address many of the concerns of Dr. Kennedy[*Reviewer #1*].

**A7**

As suggested, we repeated the experiment and indeed observed improved length of aligned read fragments. Unfortunately, sequence read yield was not improved.
