# Supplementary material for: Long read nanopore sequencing for detection of HLA and CYP2D6 variants and haplotypes
Source: F1000Res. 2015 May 20;4:17. Originally published 2015 Jan 21. [Version 2] doi: 10.12688/f1000research.6037.2 (PMC4392832; doi:10.12688/f1000research.6037.2)
Supplement: Peer review history

# Peer review: Referee response for version 2

**DOI:** 10.5256/f1000research.7015.r8715

**Published:** 2015-05-21

**Authors:** Martin Kennedy

**Affiliations:** 1 Gene Structure and Function Laboratory, Department of Pathology, University of Otago, Christchurch, New Zealand

Thanks for attending to these points. I am comfortable with the amendments that have been made.

I have read this submission. I believe that I have an appropriate level of expertise to confirm that it is of an acceptable scientific standard.

# Peer review: Referee response for version 1

**DOI:** 10.5256/f1000research.6463.r7919

**Published:** 2015-03-30

**Authors:** Thomas Hoenen

**Affiliations:** 1 Laboratory of Virology, Division of Intramural Research, National Institute of Allergy and Infectious Diseases, National Institutes of Health, Hamilton, MT, USA

This manuscript describes the analysis of three PCR-fragments generated from a human reference sample using a MinION sequencing device. Despite the preliminary nature of the data, and the fact that the paper is based on a single run of a single sample, it will nevertheless be of significant interest, and publication as a research note is in my view justified. In fact, given the broad interest in this emerging new technology, and its applicability to all life sciences (not just human (pharmaco-)genetics), the readership might be much wider than anticipated by the authors. This constitutes at the same time the biggest weakness of the paper, since it is in parts rather inaccessible for readers from fields other than human genetics (e.g. virology or microbiology), for whom this paper might nevertheless be highly relevant.

In order to address the concern that the manuscript is based on a single MinION run, and given the nature of the MAP program, it should be no problem for the authors to repeat the experiment with another sample, and while doing so specifically address the concerns of Dr. Kennedy. In addition, the authors should strive to improve accessibility to a wider audience wherever possible. Finally, it would be helpful to include additional experimental details that are currently missing.

Specific comments:

- PCR cycling conditions should be provided.
- The authors refer to the SQK-MAP003 sequencing protocol. As far as I am aware, Oxford Nanotechnologies does not make the detailed protocol available to people outside the MAP program, although there have been indications that a non-technical version of the protocol will be made available for publication purposes. The authors should reference such a protocol (including a link) as soon as possible, and approach ONT about making it available to the general public, if this hasn’t happened already.
- Did the authors perform a PCR purification prior to the library preparation? If so, what was the volume/ratio of Agencourt beads to sample?
- How did the authors extract reads from the fast5 files? Did they use poretools, or another tool (which should be referenced)?
- In general, providing more details about the exact bioinformatics workflow would be helpful.
- What was the rationale for the cut-off of 1/3 of reads for variant calling?
- It seems odd that the length of the aligned fragments from the 1D reads is so much shorter than the read length. In our hands (using a similar approach on ~2 kB PCR products amplified from virus genomes, albeit with a later chemistry/protocol version (SQK-MAP004) and using LAST for the alignment) we get much longer average alignments (92% for 2D reads, 82% for template reads, and 85% for complement reads, vs. 85%, 23% and 11% reported by the authors). This could either indicate significant advances in base-calling accuracy since the authors performed their experiments, or that their alignment is suboptimal. It might be very interesting to see whether the authors can get longer alignments using LAST or other alignment softwares in their workflow.
- It would be very helpful if the authors could repeat the experiment using the newest chemistry/protocol/software versions, which have changed considerably over the last months. At the same time this would allow them to address many of the concerns of Dr. Kennedy.

I have read this submission. I believe that I have an appropriate level of expertise to confirm that it is of an acceptable scientific standard.

# Peer review: Referee response for version 1

**DOI:** 10.5256/f1000research.6463.r7404

**Published:** 2015-02-06

**Authors:** Martin Kennedy

**Affiliations:** 1 Gene Structure and Function Laboratory, Department of Pathology, University of Otago, Christchurch, New Zealand

**General comments:**

This research note describes preliminary results from the application of a new nanopore sequencing device (the MinIon), under development by Oxford Nanopore Technologies, to the analysis of three amplicons of pharmacogenetic interest.

The paper is suitable for a research note. Although the findings are very preliminary, this is a new technology and there is a lot of interest in understanding its current capabilities, longer-term potential and limitations.

Positive aspects of the report are description of long-read nanopore sequencing on long amplicons (4-5kb), and description of data handling and bioinformatics approaches this team is using, which may aid others working with the MinIon device. Negative aspects of the report are that all the data are from only one reference sample, and one run on the device; rather than being utterly convincing,  the data suggests haplotyping on unknown samples may be possible only once error rates on the MinIon reduce; and there is an unresolved question about the CYP2D6 CNV or haplotype analyses, potentially due to sample contamination.

Despite these issues, the report is still of merit and will be of interest to many in the field.

**Specific comments:**

Methods

Long PCR has been widely used for specific amplification of CYP2D6, with  reaction conditions and primer sequences well established. It is not clear why the authors designed their own primers for this task, or how these novel primers were validated. This should be spelt out more clearly.

It would have been useful to have non-diploid control samples for the CYP2D6 CNV assay – for example a haploid (CYP2D6*5) case or multicopy case, to provide confidence that the assay was working as expected. This is relevant because of the question raised by the *2 haplotype which shows up in the MinIon analyis of NA12878.

Results

Page 6, first para: This description of the possible origins of the mystery CYP2D6*2 haplotype needs some editing for improved clarity. Not clear what is meant by “*3 and *4 duplexes forming”. Also not clear what would cause PCR biases alluded to in the last sentence of this paragraph (and of the following paragraph). 

**Typos/suggested edits:**

Abstract

MinIOn > MinIon

Should refer to NA12878 as “reference sample” rather than just “sample”

Suggest sentence be modified thus for clarity: “…statistically phased genotype data from Complete Genomics and Sequenom.”

Suggest delete “Standalone” in penultimate line.

Introduction

Suggest this sentence be changed: However, existing methods have various limitations, which may lead to adverse drug responses.  >  However, existing methods have various limitations, which may lead to failure to detect variants of pharmacogenetic significance.

Methods

Page 3, 2 ^nd^ para: indels expects > indels expected

Page 4 para 2 – clarify “The HuRef sample…”

Figure 1 title: Integrate > Integrative

Results

Page 5, para 4.  First sentence should read thus, for improved clarity: “CYP2D6 haplotype proportions in MinIon data were identified by interrogating clinical marker positions…”

Supp File S1

It would be helpful to indicate which of the sequences is CYP2D6 and which is CYP2D7.

I have read this submission. I believe that I have an appropriate level of expertise to confirm that it is of an acceptable scientific standard.
